# Novel prediction model of early screening lung adenocarcinoma with pulmonary fibrosis based on haematological index

**DOI:** 10.1186/s12885-024-12902-6

**Published:** 2024-09-27

**Authors:** Haiyang Li, Xing Fu, Mingtao Liu, Jiaxi Chen, Wenhan Cao, Zhiman Liang, Zhangkai J. Cheng, Baoqing Sun

**Affiliations:** 1 Department of Clinical Laboratory, StateKey Laboratory of Respiratory Disease, National Center for Respiratory Medicine, National Clinical Research Center for Respiratory Disease, First Affiliated Hospital of Guangzhou Medical University, Guangzhou Institute of Respiratory Health, Guangzhou, 510120 China; 2 MRC Biostatistics Unit, University of Cambridge, Cambridge, CB2 0SR UK; 3 Fudan University School of Life Sciences, Fudan University, Shanghai, 200433 China; 4 KingMed School of Laboratory Medicine, Guangzhou Medical University, Guangzhou, 511495 China; 5 Guangzhou Laboratory, Guangzhou, 510320 China

**Keywords:** Lung adenocarcinoma, Pulmonary fibrosis, Bayesian model, Serum biomarkers, Machine learning

## Abstract

**Background:**

Lung cancer (LC), a paramount global life-threatening condition causing significant mortality, is most commonly characterized by its subtype, lung adenocarcinoma (LUAD). Concomitant with LC, pulmonary fibrosis (PF) and interstitial lung disease (ILD) contribute to an intricate landscape of respiratory diseases. Idiopathic pulmonary fibrosis (IPF) in association with LC has been explored. However, other fibrotic interrelations remain underrepresented, especially for LUAD-PF and LUAD-ILD.

**Methods:**

We analysed data with statistical analysis from 7,137 healthy individuals, 7,762 LUAD patients, 7,955 ILD patients, and 2,124 complex PF patients collected over ten years. Furthermore, to identify blood indicators related to lung disease and its complications and compare the relationships between different indicators and lung diseases, we successfully applied the naive Bayes model for a biomarker-based prediction of diagnosis and development into complex PF.

**Results:**

Males predominantly marked their presence in all categories, save for complex PF where females took precedence. Biomarkers, specifically AGR, MLR, NLR, and PLR emerged as pivotal in discerning lung diseases. A machine-learning-driven predictive model underscored the efficacy of these markers in early detection and diagnosis, with NLR exhibiting unparalleled accuracy.

**Conclusions:**

Our study elucidates the gender disparities in lung diseases and illuminates the profound potential of serum biomarkers, including AGR, MLR, NLR, and PLR in early lung cancer detection. With NLR as a standout, therefore, this study advances the exploration of indicator changes and predictions in patients with pulmonary disease and fibrosis, thereby improving early diagnosis, treatment, survival rate, and patient prognosis.

**Supplementary Information:**

The online version contains supplementary material available at 10.1186/s12885-024-12902-6.

## Introduction

Lung cancer (LC) emerges as a relentless and life-threatening condition, manifesting in the unbridled proliferation of lung cells. This insidious disease is the most frequently identified cancer across the globe and the forefront cause of cancer-related mortality [1, 2]. The two main subtypes of lung cancer are non-small cell lung cancer (NSCLC) and small cell carcinoma (SCLC). NSCLC includes adenocarcinoma (LUAD) and squamous cell carcinoma, and large cell carcinoma [3], with lung adenocarcinoma being the most common [4]. Pulmonary fibrosis (PF) is a scarring process that amplifies cancer risk, with the enigmatic role of interstitial lung disease (ILD) weaving a more complex pattern [5]. Interstitial lung diseases (ILD) — which include a wide variety of conditions, among them idiopathic pulmonary fibrosis (IPF) — also increase the risk of developing lung cancer [6]. The association between interstitial lung disease (ILD) and lung cancer was first suggested in 1939 [7], and since then, the role of pulmonary fibrosis in tumour formation and development has been extensively investigated. Several reviews have reported an increased risk of lung cancer in individuals with ILD [8, 9]. Also, some research on lung cancer with interstitial lung disease (LC-ILD) has been conducted [10, 11]. Chen *et al.* [12] conducted research with a sample of 13,085 hospitalized LC patients and demonstrated that most LC-ILD patients were diagnosed with adenocarcinoma (LUAD-ILD), even though the LC-ILD only constituted a small percentage (3.89%) of the sample. However, most of the common studies are about lung cancer combined with idiopathic pulmonary fibrosis, and few have studied lung cancer combined with other forms of pulmonary fibrosis beyond IPF. Therefore, prognostic research in lung cancer, particularly LUAD-PF and LUAD-ILD is vital for guiding personalized treatments, enhancing patient outcomes and offering insights into disease progression and survival rates.

Early diagnosis of lung disease is particularly important for treatment. The conventional technology for early detection of lung disease is computed tomography (CT). However, it has a higher risk of false positives, which may lead to overdiagnosis and unnecessary treatment [13, 14]. Therefore, non-invasive or minimally invasive biomarkers are crucial for complementing existing CT scans in early detection, predicting disease recurrence and assessing prognosis. For instance, emerging liquid biopsy-derived (such as blood-based) biomarkers have been proven highly effective in identifying lung cancer biomarkers [15]. Society *et al.* [16] have summarized the current knowledge on interstitial lung disease serum biomarkers and their potential value as prognostic and diagnostic tools. Such as serum albumin (ALB) and globulin (GLB) [17, 18], lactate dehydrogenase (LDH) [19], and electrolytes [20] are widely used as predictive indicators for lung cancer. Inflammation, detectable through serum biomarkers, is critical for cancer progression and mirrors the host’s immune status [21]. And some inflammatory biomarkers such as C-reactive protein (CRP) [22], carcinoembryonic antigen (CEA), neuron-specific enolase (NSE) [23], red cell distribution width (RDW) [24]. While these indicators have proven useful, no existing studies have combined them to predict and analyse the progression of lung cancer.

Traditional clinical diagnosis for patients with ILD or LC concurrent pulmonary fibrosis, typically through high- resolution computed tomography (HRCT) and transbronchial lung biopsy (TBB) [25, 26], is greatly influenced by the physician’s clinical experience and subjectivity. Consequently, In order to gain a more accurate and rapid diagnosis for different lung diseases, researchers have begun to focus on the prediction of lung cancer through serum indicators [27–29]. Ramos et al. [30] probes the prognostic potential of systemic inflammatory response markers in resectable lung cancer, with albumin-to-globulin ratio (AGR), neutrophil-to-lymphocyte ratio (NLR) and platelet-to-lymphocyte ratio (PLR) and emerging as key indicators. Meanwhile, Shoji et al. [31] pointed out that monocyte-to-lymphocyte ratio (MLR) level is the best predictive indicator for lung cancer recurrence Cannon et al. [32]finds preoperative MLR an independent prognostic indicator for survival and recurrence-free outcomes in early-stage lung cancer post-surgery. Watase et al. [33] highlights the NLR as a key biomarker for diagnosing and predicting the prognosis of ILD-PF, indicating its potential utility in patient treatment. The integration of systemic inflammatory markers, detailed diagnostic criteria, and a multidisciplinary approach to diagnosis and management, enriches the understanding and prognostication of lung cancer, especially in the presence of complex conditions such as ILD-PF and LUAD-PF. This comprehensive strategy ensures that treatment is tailored to the unique needs of each patient, potentially improving outcomes in this challenging patient population.

**Fig. 1:**
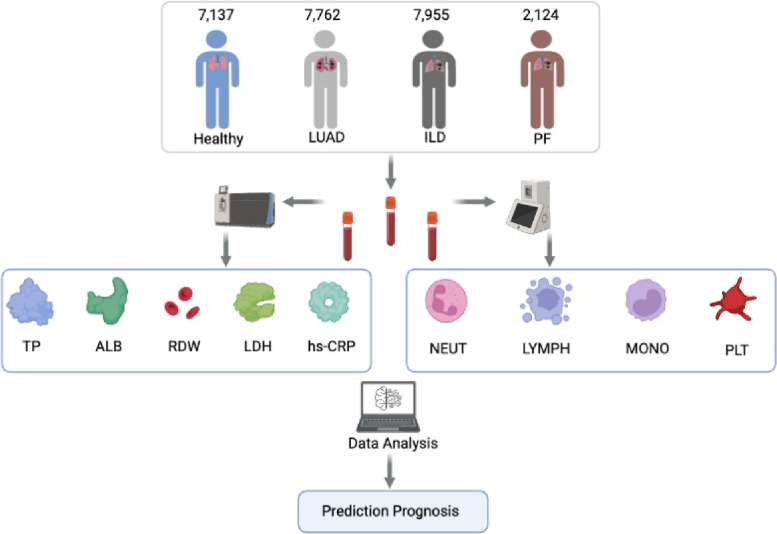
The framework of analysis and prediction of indexes of LUAD, ILD, and cases involving both complex PF. The blood samples were collected from a diverse group of individuals, including 7,762 patients diagnosed with LUAD, 7,955 individuals suffering from ILD, approximately 2,124 patients with complex PF (including 615 patients with LUAD-PF, 1228 patients with ILD-PF, and 281 patients with LUAD-ILD-PF), and a control group of around 7,137 healthy subjects. Following collection, these samples were subjected to advanced biochemical analysis to identify a variety of serum and inflammatory biomarkers. The derived data were then incorporated into a dedicated prediction model, expressly designed to systematically predict both the diagnosis of these pulmonary conditions

Here, we collected 17,841 participants with different types of lung diseases and 7,137 healthy individuals to test routine biochemical indices and specific indicators, as shown in Fig. 1. After that, we trained a naive Bayesian machine learning algorithm to optimize our prediction model through the collected blood indexes of the subjects and show the prediction performance of the model through the Receiver Operating Characteristic (ROC) curve and the area under the curve (AUC). Additionally, correlation analysis was conducted between subtype indicators to identify the performance of serum indicators in different lung diseases. Finally, serum indicators, electrolytes and inflammatory biomarkers data were combined to predict and diagnose lung diseases more accurately and quickly through statistical models and machine learning algorithms.

## Methods

### Participants

**Fig. 2:**
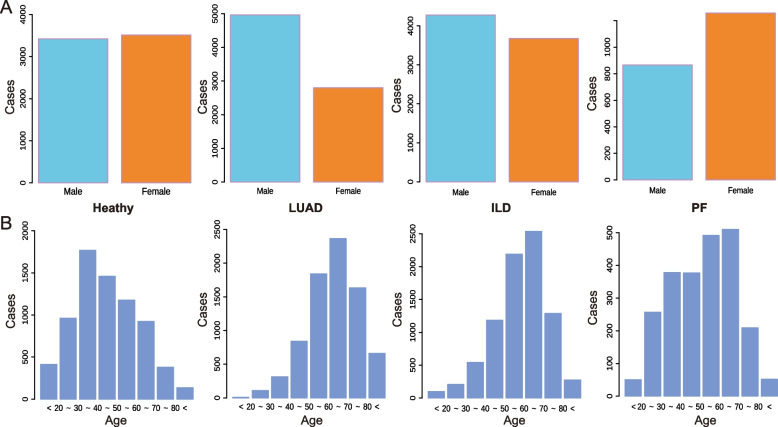
Basic information and index analysis of participants. **A** The sexual distribution of the healthy population, LUAD, ILD, and complex PF patients. **B** The age distribution of participants

This study’s samples comprised 7,137 healthy individuals, 7,762 patients with LUAD, 7,955 patients with ILD, and 2,124 patients with complex PF (including 615 patients with LUAD-PF, 1228 patients with ILD-PF, and 281 patients with LUAD-ILD-PF), which were collected from the First Affiliated Hospital of Guangzhou Medical University over a ten-year period from June 2013 to June 2023. Venous blood samples were collected from participants, all of whom had been medically tested and radiologically confirmed to be diagnosed with either LUAD, complex PF, ILD, or all. Heparin was used as an anticoagulant in the collection process. These samples were gathered at 4^◦^C, and centrifuged at 3000 rpm within 30 minutes, and the supernatants were then stored in a cleansing pack and kept at -80^◦^C. Additionally, a random selection of 7,137 blood specimens from healthy individuals was chosen as the control group. And the gender distribution of total participants with specifics detailed in Fig. 2. Moreover, the gender and age distribution of complex PF subgroups were demonstrated (Supplementary Fig. 3A, B, C, D). Participation in the study was based on voluntary informed consent from all individuals involved, receiving approval from the Ethics Committee of the First Affiliated Hospital of Guangzhou Medical University’s Scientific Research Project Reviews (Document No.136 of 2022) and in strict compliance with the guidelines of the Declaration of Helsinki.

### Patients’ data acquisition

We meticulously gathered the biochemical data from patients’ blood, which incorporated indicators like ALB, total protein (TP), blood platelet (PLT), LDH, RDW, along with inflammation markers such as CRP, neutrophil (NEUT), lymphocyte (LYMPH), and monocyte (MONO). These evaluations were executed via the Automatic Biochemical Analyser LABOSPECT 006 (Hitachi, Ltd, Tokyo, Japan). In parallel, the count of the patients’ granulocytes was determined using the Coulter AcT 5diff AL (Autoloader) Hematology Analyser (Beckman Coulter, Ltd, USA).

### Statistical analyses

The diagnostic analysis with the correlation coefficient, loess regression, the Kruskal–Wallis test. The correlation coefficients, which are statistical measures of the strength and direction of the linear relationship between two variables, were employed for all blood index calculations and analyses. This coefficient ranges from -1 to 1, where 1 denotes a perfectly positive correlation, and vice versa, besides, values closer to 0 represent weaker correlations. Loess regression, also known as locally weighted regression, which is a statistical technique that partitions the samples into local neighbourhoods. Within each neighbourhood, the samples are assigned fixed but non-parametric weights, which are used to construct a regression curve. These individual regression curves are then combined to form the overall regression curve [34]. The Kruskal-Wallis test is a non-parametric statistical method used to compare the medians of two or more independent samples with equal or different sample sizes. The test allows researchers to determine if there are significant differences in the distributions of the samples based on their medians. Naive Bayes classifiers are simple probabilistic models based on Bayes’ theorem, assuming independence between features [35]. Additionally, the ROC curve was implemented to assess and compare the efficacy of the diagnostic models and ascertain their practical applicability [36, 37]. Visualization of the ROC curve and its Area Under the Curve (AUC) was facilitated by the *pROC* package. The AUC is a critical metric within the ROC curve, used to evaluate if positive outcomes are ranked above negative ones. All biochemical and blood indicators were examined using an ROC curve, with those demonstrating higher AUC indexes chosen for subsequent analysis.

### Modelling of predictive models

Our model uses Bayes’ theorem for classification (based on Supplementary Fig. 8) and assumes that the classification is predictor-independent. It assumes that the naive Bayes classifier in the presence of a particular feature in a class is independent of any other feature when the naive Bayes model is suitable for the establishment and further analysis of very large data sets. This model is a very simple and complex classification method, which can be well-classified even in complex scenes. In this study, 23 serum-related indicators were normalized, and the data were divided by random sampling. Therefore, we divided the healthy people and patients with lung diseases into a training set (70%) and a verification set (30%). We collect the results and select the model with the best performance, meanwhile, the prediction accuracy is measured on the test set. Then, the model is optimized for the number of variables selected for each tree. In the process of adjusting the parameters, cross-validation was used to prevent overfitting of the Bayes model and to maintain the stability and practicality of the model. Discrimination performance was assessed based on the ROC curve and the corresponding AUC value.

### Data visualization

All of the data acquisition and data statistical analyses were performed using R core team version 4.2.1 and Python 3.7. Otherwise, optimization of colour and typesetting is completed through Adobe Illustrator (https://www.adobe.com). The basic column chart and box diagram are drawn by the "matlibplot" Python package (version 3.5). The visualization of the pair plots was performed through the "seaborn" python package (version 0.11.2; https://seaborn.pydata.org/). Our model was established in R by naivebayes package (version 0.9.7; https://github.com/majkamichal/naivebayes). A heat scatter was created using the "LSD" package (version 4.1-0; https://cran.r-project.org/web/packages/LSD) in R. The index differ- ences between male and female medical records of different lung diseases were visually compared by "beanplot" package (version 1.3.1; https://cran.r-project.org/web/packages/beanplot/). The correlation coefficients between the data indices in the study were visualized by "ggcorrplot" package (version 0.1.3; https://cran.r-project.org/web/packages/ggcorrplot/).

## Results

### The demographic characteristics of participants

In our study, we observed that the predominant age range for the healthy population centres around 40 ± 15 years. The detailed distribution of patient categories can be found in Table S1. Our findings highlight that patients diagnosed with LUAD have an average age of 63 ± 13 years. In comparison, ILD patients average 58 ± 15 years, while the complex PF patients (those with concurrent LUAD and ILD) present an average age of 56 ± 16 years, placing them within a similar age bracket.

Interestingly, the proportion of male patients diagnosed with all types of lung diseases significantly overshadows that of females, a trend that is apparent in this study. However, it is noteworthy that a higher proportion of patients diagnosed with PF, which combines LUAD and ILD, are women, as opposed to men. The age distribution of participants is graphically represented in Fig. 2, which depicts a histogram illustrating the age distribution of patients with LUAD, ILD, and complex PF, corresponding to the statistical result presented in Table S1. The peak age range for healthy individuals is observed to be between 30 and 40 years old. Conversely, the peak age range for patients diagnosed with LUAD and ILD is concentrated in the 60 to 70 age group. Although the general trend of age distribution is similar between the two groups, it is worth noting that the proportion of ILD patients in the 50 to 60 age group shows an increasing trend toward younger ages. Notably, the distribution of patients with complex PF differs significantly from that of patients with LUAD and ILD, demonstrating a tendency towards a younger age demographic.

In detail, we divided the complex PF group into different complications, which totally contained 2124 patients with LUAD or ILD complicated with PF, including 615 patients with LUAD-PF, 1228 patients with ILD-PF, and 281 patients with LUAD-ILD-PF (L-I-PF). The distribution of complex PF patients depicts that a higher proportion of patients with complex PF are female, especially in ILD-PF patients (Supplementary Fig. 3A). Furthermore, the peak age range for patients with LUAD-PF is observed to be between 60 and 70 years old (Supplementary Fig. 3B), and the same age range is observed for L-I-PF patients (Supplementary Fig. 3D). Conversely, for patients afflicted with ILD-PF, the pinnacle of the age distribution is concentrated within the range of 40 to 50 years (Supplementary Fig. 3C).

### Analysis of blood biochemical indicator

**Fig. 3:**
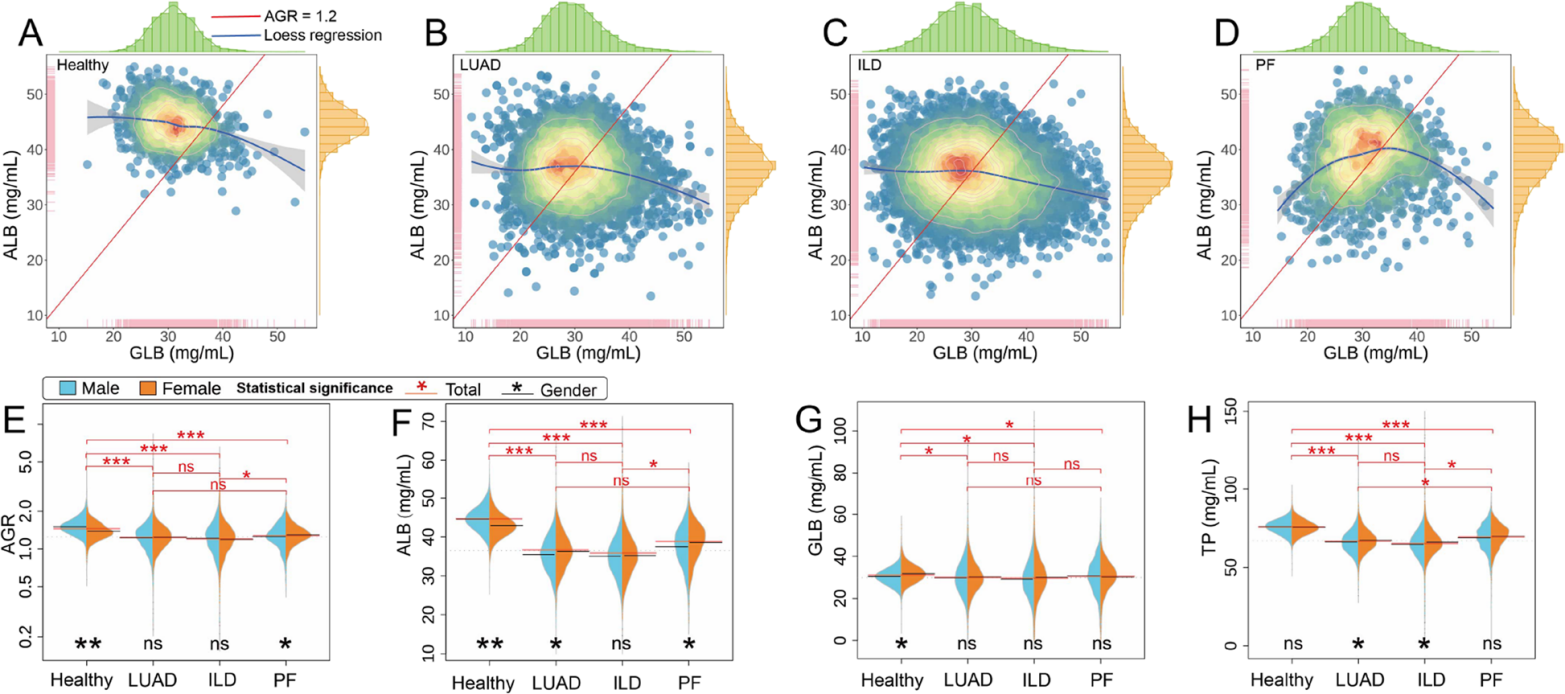
The ALB/GLB density scatters plot of (**A**) healthy population, (**B**) LUAD, (**C**) ILD and (**D**) complex PF patients. The beanplot of indicators distribution between genders with (**E**) AGR, (**F**) ALB, (**G**) GLB and (**H**) TP

Protein indicators are highly sensitive markers in the human body, following illness, these indicators undergo significant changes [17]. Similarly, the AGR has been employed as a prognostic factor for lung cancer patients by Li et al. [38]. We conducted an analysis of the distribution of ALB/GLB among different participants, as depicted in Fig. 3A, B, C, D. The ALB content in healthy individuals primarily concentrates at 45 mg/mL, whereas LUAD and ILD patients exhibit comparatively lower levels at 40 mg/mL. Likewise, the GLB levels in the patient groups are the same as those in healthy individuals, signifying a significant disparity between healthy and lung diseases. The AGR ratio serves as a valuable health indicator, with ratios higher or lower than 1.2 often indicating potential health concerns [39]. Gaining an understanding of these ratios can provide key insights into an individual’s health status and guide potential interventions. Therefore, maintaining a balanced protein profile is crucial for optimal health. Based on the results obtained from Fig. 3A, B, C, D (where the red line represents AGR = 1.2) and Table S1, it is evident that the AGR of healthy individuals is predominantly concentrated above 1.2, while the AGR median (IQR) of patients is close to 1.2 and the scatter of it tend to fall below 1.2 except complex PF.

Since variations in indicator levels may exist between males and females, we segregated the data into male and female groups for further analysis. We assessed the statistical significance of each indicator within different participant groups and genders. The statistical significance was denoted as follows: "***" indicates significance with *p* < 0.001; "**" indicates significance with *p* < 0.01; "*" represents significance with *p* < 0.05, and "ns" signifies non-significance. In the gender-based analysis, the results indicated significant differences between male and female indicators in the healthy population, while there were no substantial statistical differences observed within the patient group. Moreover, in the analysis of participant groups, extremely significant differences were found between healthy individuals and those with LUAD, ILD, and complex PF, except for the GLB index. However, no statistical difference was observed between LUAD and ILD.

### Analysis of the ratio between blood indicators

**Fig. 4:**
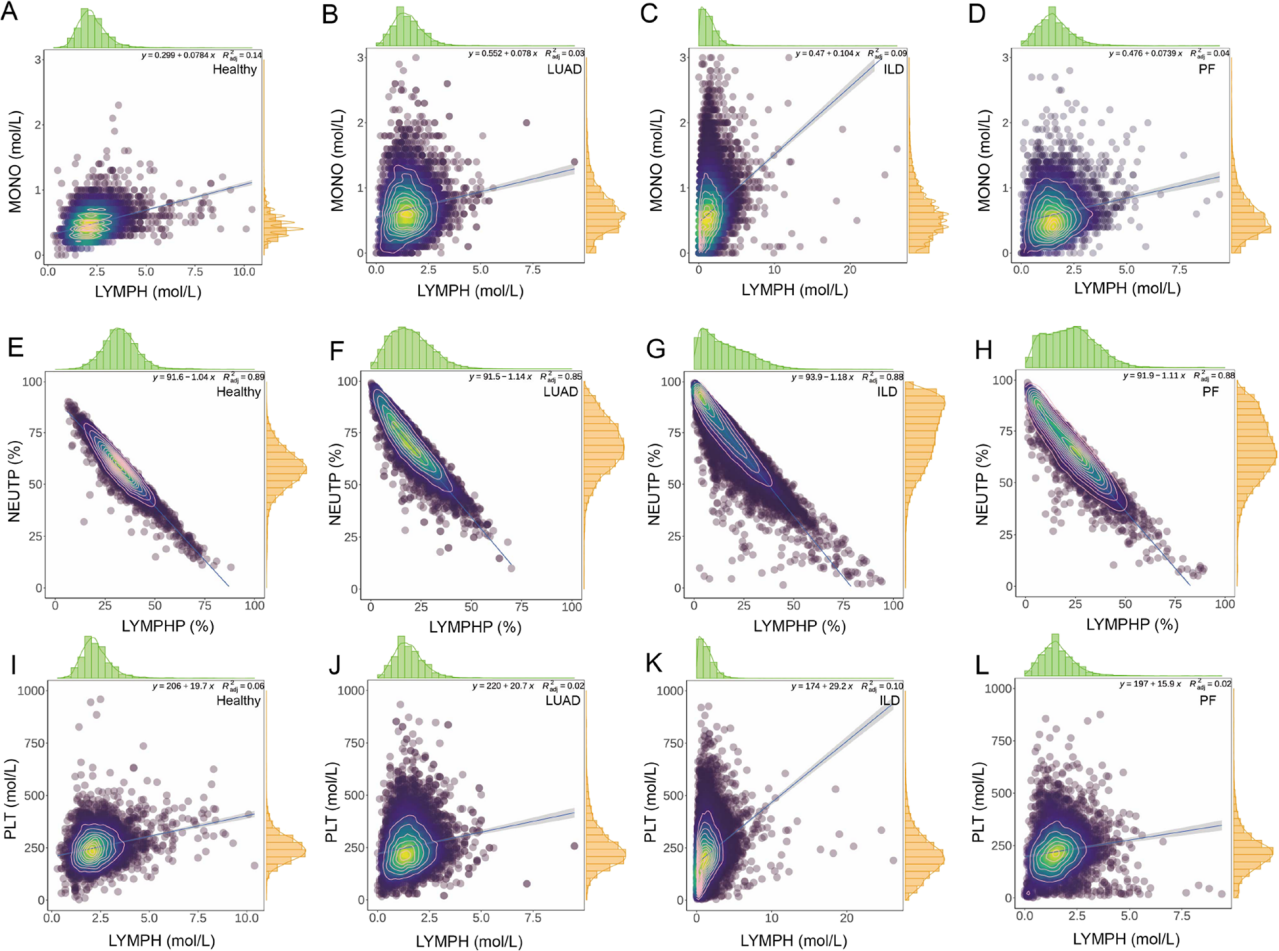
The MONO/LYMPH density scatters plot of (**A**) healthy population, (**B**) LUAD, (**C**) ILD and (**D**) complex PF patients. The blood indicators of NEUTP/LYMPHP in (**E**) healthy population, (**F**) LUAD, (**G**) ILD and (**H**) complex PF patients. The distribution of PLT/LYMPH scatters in the (**I**) healthy population, (**J**) LUAD, (**K**) ILD and (**L**) complex PF patients

The ratio of blood indices holds valuable significance for clinical diagnosis, providing guidance in previous studies. These ratios serve as intuitive indicators of disease severity and prognostic effectiveness. In our study, we compared various LYMPH-related ratios, such as MONO/LYMPH, neutrophil proportion /lymphocyte proportion (NEUTP/LYMPHP), and PLT/LYMPH, among different participants. To analyse the distribution of MONO and LYMPH, we examined the density scatters depicted in Fig. 4A, B, C, and D. These figures demonstrate a linear relationship between increasing LYMPH and MONO counts. The slopes of the fitting equations for the healthy population, LUAD, ILD, and complex PF were determined as 0.0784, 0.078, 0.104, and 0.0739, respectively. However, it is worth noting that the corresponding *R*^2^ values are relatively small among all groups, including LUAD, ILD, complex PF patients, and the healthy population. Moreover, we examined the distribution of NEUTP/LYMPHP scatters in Fig. 4E, F, G, and H, which displayed a strong linear relationship with higher *R*^2^ values in the groups of healthy population, LUAD patients, ILD patients, and complex PF patients. The *R*^2^ values for the healthy population, LUAD, ILD, and complex PF are 0.89, 0.85, 0.88, and 0.88, respectively. After conducting a linear fit, we determined the slopes of the fitting equations to be -1.04 for healthy individuals, -1.14 for LUAD patients, -1.18 for ILD patients, and -1.11 for complex PF patients. It is evident that the slope of the healthy population is greater than that of the lung patients. Additionally, among the lung diseases, the slope of the complex PF patients is the highest compared to the other patient groups. Based on the scatter density heat analysis, we observed that healthy individuals are predominantly concentrated around the midpoint of the ratio distribution. In contrast, lung patients exhibit density hotspots that are concentrated in the higher NEUTP area. Likewise, we analysed the distribution of NEUTP/LYMPHP scatters, which also depicted a strong linear relationship with higher *R*^2^ values in the complex PF subgroups (Supplementary Fig. 4). The *R*^2^ values for the complex PF subgroups of LUAD-PF, ILD-PF, and L-I-PF are 0.86, 0.92, and 0.87, respectively. The scatter density hotspots of the patients with LUAD-PF, ILD-PF, and L-I-PF are concentrated in the higher NEUTP area than the LYMPHP area with a Y-axis intercept of 92.3, 94.3, and 93.2, respectively.

Lastly, we investigated the scatter distribution of PLT/LYMPH, which serves as an inflammatory biomarker reflecting an individual’s immune condition. The relationship between platelets and lymphocytes was illustrated in Fig. 4I, J, K, and L, exhibiting a linear relationship. The slopes for the healthy population, LUAD, ILD, and complex PF were found to be 19.7, 20.7, 29.2, and 15.9, respectively. Notably, the *R*^2^ values are considerably small both for healthy individuals and those with lung diseases. The density hotspots for both PLT and LYMPH in healthy individuals and lung patients are concentrated in the lower range.

### The statistical significance of participants

Statistical significance analysis is a reliable approach for comparing differences among groups of samples. In our examination of indicator distributions, we assessed the significance of differences between these groups. Fig. 3E illustrates the distribution of each indicator in the AGR ratio, along with a statistical analysis of these differences. This analysis includes comparisons between male and female indicators, as well as differences across participant groups.

**Fig. 5:**
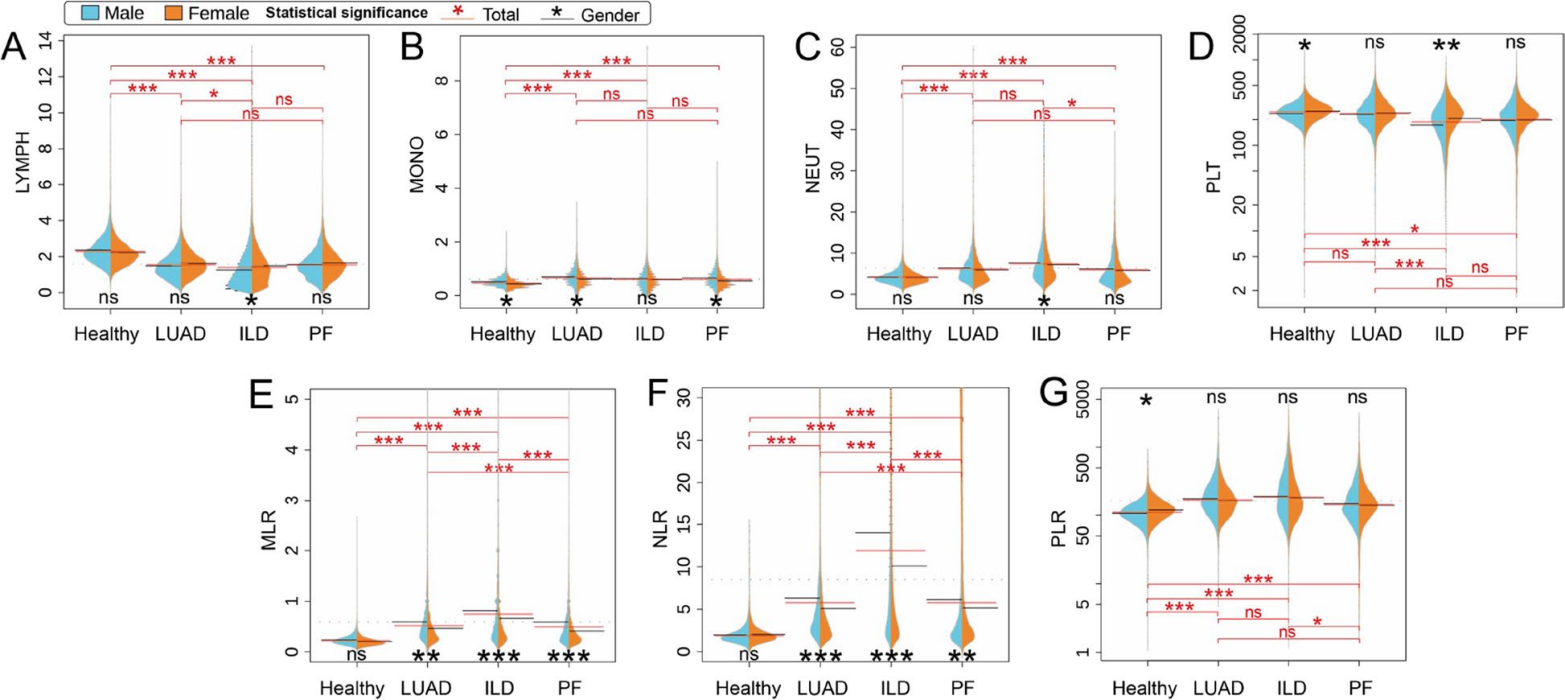
The beanplot of indicators distribution between gender with (**A**) LYMPH, (**B**) MONO, (**C**) NEUT, (**D**) PLT, (**E**) MLR, (**F**) NLR, and (**G**) PLR

We conducted single indicator analyses in the group of healthy individuals, LUAD patients, ILD patients, and complex PF patients, including LYMPH (Fig. 5A), MONO (Fig. 5B), NEUT (Fig. 5C), and PLT (Fig. 5D), as well as ratios of certain inflammatory biomarkers, such as MLR (Fig. 5E), NLR (Fig. 5F), and PLR (Fig. 5G), between male and female samples. The statistical differences of these single indicators, particularly LYMPH, MONO, and NEUT, between the healthy population and lung patients, exhibit notable significance. However, when comparing males and females, the statistical differences are less pronounced. Notably, the indicator of LYMPH demonstrates statistical significance between LUAD and ILD patients, while the indicator of NEUT shows significance between ILD and complex PF patients. However, the indicator of MONO does not demonstrate significance in the three lung diseases. Moreover, the PLT indicator displays statistical significance between healthy individuals and ILD patients, as well as between LUAD and ILD. And the PLT indicator also shows a statistical significance between males and females in both healthy participants and ILD patients. Additionally, we analysed the differences in biomarker ratios, namely MLR, NLR, and PLR, between healthy individuals and lung patients. These ratios show high statistical significance between them. Particularly, even when considering gender differences, MLR and NLR demonstrate significant differences across LUAD, ILD, and complex PF. Conversely, the PLR indicator does not exhibit any substantial significance in relation to gender among LUAD, ILD, and complex PF but shows a statistical significance between ILD and complex PF participants.

Additionally, we analysed the single indicators detailed including ALB, MONO, LYMPH, NEUT, hs-CRP and PLT, in healthy individuals and complex PF subgroups of LUAD-PF, ILD-PF, and L-I-PF patients (Supplementary Fig. 3). These indicators demonstrate statistical differences between the healthy population and complex PF subgroups, except for the indicator MONO, which does not show significant differences between healthy individuals and ILD-PF patients. In the complex PF subgroup of LUAD-PF and ILD-PF patients, these indicators of ALB, MONO, LYMPH, NEUT, and hs-CRP show statistical differences between them. Comparing the patients with LUAD-PF and L-I-PF, only the ALB and hs-CRP indicators demonstrated noteworthy statistical differences. In the ILD-PF and L-I-PF subgroups, with the exception of the NEUT and PLT indicators, the indicators ALB, MONO, and LYMPH displayed substantial statistical disparities.

### Interrelation between blood indicators

We selected a total of 23 blood index indicators for the four groups: the healthy population, LUAD patients, ILD patients, and complex PF patients. These indicators include high-sensitivity C-reactive protein (hs-CRP), CRP, NEUT, white blood cell count (WBC), neutrophil proportion (NEUTP), and lymphocyte proportion (LYMPHP), among others. After conducting the correlation analysis of these indicators (Supplementary Fig. 1), the highest correlations were found between hs-CRP and CRP, NEUT and WBC, NEUTP and NLR, and LYMPHP and LYMPH in all of the four groups. Conversely, there was a strong negative relationship between LYMPHP and NLR, and between NEUTP and LYMPHP of different groups. The correlation relationship between LYMPHP and NLR extremely followed the equation rules of NLR = NEUTP/LYMPHP, in which correlation coefficients in groups of the healthy population, LUAD, ILD, and complex PF patients are -0.92, -0.94, -0.95, and -0.94 respectively. However, the LYMPHP showed a little weak negative correlation with MLR and PLR, which correlation coefficients of -0.54, and -0.45 in patients with complex PF, respectively. Moreover, one of the highest correlations between hs-CRP and CRP in groups of the healthy population, LUAD, ILD, and complex PF are 1.0, 0.61, 0.94, and 0.93, respectively, which may provide some reference values for the research of other projects. Further, besides the general and mathematic-related correlations, there are some other correlations. Between ALB and calcium (Ca), the correlation coefficients are 0.31, 0.36, 0.47, and 0.52 in the healthy population, LUAD, ILD, and complex PF patients, respectively, which display an increasing trend. The correlation between neuron-specific enolase (NSE) and hs-CRP is 0.31, 0.09, 0.07, and 0.11 in the four types of lung diseases, notably that the correlation coefficient in healthy people is higher than in others. Moreover, the correlation coefficients between LDH and NEUTP, LDH and CEA, and LDH and AGE in ILD and complex PF respectively are positive correlations. However, in healthy people, the correlation coefficients between LDH and NEUTP, LDH and CEA, and LDH and AGE show negative or weak correlations. Obviously, the correlation between AGR and CRP is -0.23, -0.25, -0.32, and -0.15 in the healthy population, LUAD, ILD, and complex PF patients respectively.

Moreover, we conducted an analysis of scatter and density distributions between different indicators. The distributions between LYMPHP and NLR, LYMPHP and MLR, as well as LYMPHP and PLR, exhibited an inverse proportion function (Supplementary Fig. 2). These distributions followed the negative correlation relationships (Supplementary Fig. 1). Additionally, the distributions of NLR, MLR, and PLR displayed an increasing trend with NEUTP, indicating a strong correlation between NEUTP and these indicators, particularly NLR. Moreover, interesting distributions were observed between NLR and NEUT, as well as between PLR and PLT, illustrating a linear relationship between these indicators.

### Performance of a predictive model for lung patients

Here, we constructed and trained a predictive model to predict the risk of lung diseases. The ROC curve is a visual tool that effectively demonstrates the relationship between sensitivity and (1-specificity) at different thresholds [37]. The AUC serves as a numerical measure to assess the overall performance of the ROC curve. In this study, we utilized this approach to analyse the predictive capabilities of various ratios, namely AGR, MLR, NLR, and PLR, with the aim of determining the most effective predictor.

**Fig. 6:**
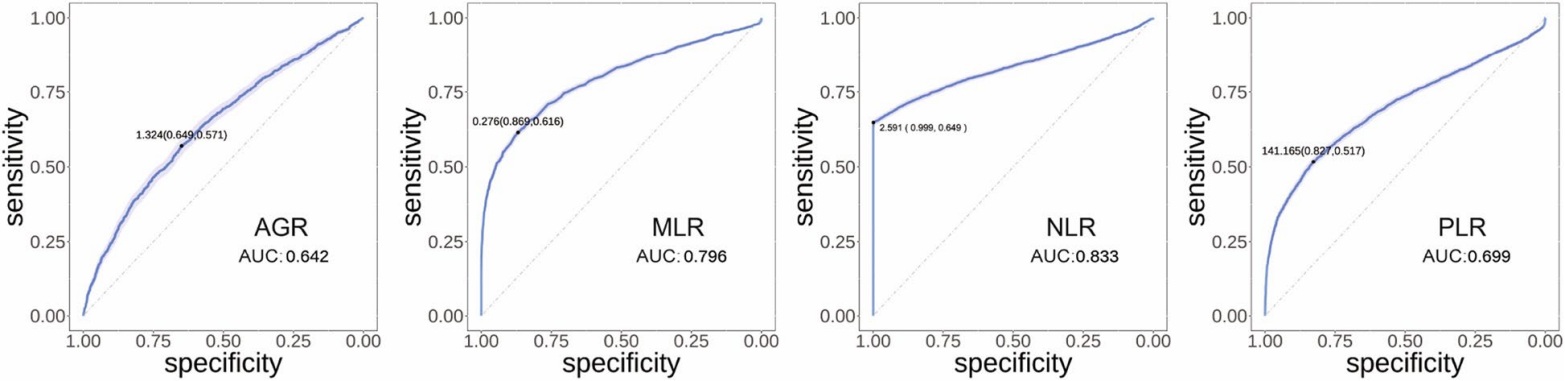
The performance of predictive model test results for the ratio of indicators such as AGR, MLR, NLR, and PLR in healthy people and complex PF patients

Figure 6 presents the grouped ROC curve results of these four significant single indicator ratios of complex PF patients in the predictive model. The lighter colour represents the 95% confidence interval of the model’s predictive performance. Notably, among the four indicators, NLR displayed the highest accuracy, with an average index of 2.591, a sensitivity of 0.999, a specificity of 0.649, and an AUC of 0.833. The other indicators, such as AGR, MLR, and PLR, also exhibited favourable predictive abilities, albeit with slightly lower performance, as indicated by their respective AUC values of 0.642, 0.796, and 0.699.

Additionally, we conducted an examination of prediction performance in complex PF patients using single indicators such as ALB, TP, NEUT, LYMPH, MONO, PLT, NEUTP, and LYMPHP (Supplementary Fig. 5). Notably, the indicator LYMPHP exhibited superior predictive ability compared to other indicators, with an average index of 25.55 %, a sensitivity of 0.978, a specificity of 0.667, and an AUC of 0.843. The second most effective predictive indicator was NEUTP, with an AUC of 0.804. Conversely, the MONO indicator displayed the weakest predictive capability, with an average index of 0.605 mol/L, a sensitivity of 0.869, a specificity of 0.321, and an AUC of 0.598.

Moreover, in the assessment of predictive performance for patients with ILD and LUAD, the efficacy of four distinct single indicator ratios was examined (Supplementary Fig. 6). Specifically, in the context of healthy individuals and LUAD patients (Supplementary Fig. 6A), both MLR and NLR manifested good predictive power, represented by AUC values of 0.775 and 0.758, respectively. Contrarily, the AGR and PLR exhibited weaker predictive capacities with AUC values of 0.628 and 0.69. In a separate examination involving healthy individuals and ILD patients (Supplementary Fig. 6B), AGR and PLR continued to show limited predictive strength, with AUC values of 0.531 and 0.529, while MLR and NLR maintained their good predictive attributes, indicated by AUC values of 0.602 and 0.609, respectively. These findings emphasize the utility of these indicators in predictive modelling, although their effectiveness may vary depending on the specific patient population.

## Discussion

In this study, we identified several serum biomarkers associated with the diagnosis of lung diseases (LUAD, ILD, PF) through statistical data analysis. Over a span of ten years, we collected data from 7,137 healthy individuals, 7,762 LUAD patients, 7,955 ILD patients, and 2,124 patients with complex PF. Interestingly, the distribution of male patients diagnosed with all types of lung diseases exceeds that of females (Fig. 2). However, in female patients diagnosed with PF, the proportion of females outweighed that of male patients, particularly in ILD-PF patients (Supplementary Fig. 3A). Moreover, to identify blood indicators related to lung diseases and their complications, and to compare the relationships between different indicators and lung diseases, we successfully applied the naive Bayes model for biomarker-based prediction of patients’ diagnoses. This approach demonstrated high accuracy.

The AGR, which combines two independent prognostic indicators, has been identified in prior research as a significant predictor of survival outcomes in various cancers such as breast cancer [40], kidney cancer [41], leukemia [42], and lung cancer [18, 43]. Our investigation has further revealed a marked disparity in the AGR between healthy individuals and those diagnosed with LUAD combined with PF and/or ILD in Fig. 3 and Supplementary Fig. 3. While the AGR of healthy individuals is predominantly above the critical threshold of 1.2, those suffering from LUAD, ILD, and complex PF generally fall below this level. This observation supports the threshold suggested by He et al. [44], reinforcing the potential health risks associated with an AGR that deviates from 1.2. Interestingly, these findings align with those of Suh et al. [45], who provided a detailed analysis of cancer incidence in relation to AGR in generally healthy adults, suggesting a higher probability of cancer incidence when AGR is below 1.0. However, for combined diseases such as PF, the AGR as an independent predictor, does not distinguish well between these patient groups, suggesting a need for further investigation into multi-morbidity prognosis. Besides analysing the scatter distribution of ALB and GLB, we also assessed the statistical significance of AGR in different participant groups and found that there were extremely significant differences between healthy individuals and those with LUAD, ILD, and complex PF. Thus, AGR could significantly predict the presence of lung disease, differentiating patients from healthy individuals. Nevertheless, further research is needed to utilize AGR in distinguishing between different types of lung diseases [46].

Furthermore, we analysed individual indicators including LYMPH, MONO, NEUT, and PLT, and inflammatory ratios such as MLR, NLR, and PLR, which have been consistently validated for their prognostic values across both operable and advanced inoperable cancers [47]. The scatter density heat map for healthy individuals primarily concentrates around the midpoint, whereas lung patients exhibit density hotspots concentrated in the regions with higher NEUTP values (Fig. 4E, F, G, D). Figure 5F reveals a higher level of NLR in patients with LUAD, ILD, and complex PF when compared to healthy individuals, which is consistent with the distribution observed for both NEUTP and LYMPHP in the scatter density heat maps. The PLR also shows a higher level of lung diseases than in healthy individuals (Fig. 5G). These results support that high NLR and high PLR are significantly predictive of poorer overall survival [48]. Zhu *et al.* [49] demonstrated that NLR and PLR alone exhibit moderate performance in distinguishing lung cancer patients from healthy subjects. This observation suggests a systemic inflammatory response associated with lung cancer, where neutrophil and platelet counts increase relative to lymphocyte counts, potentially due to tumour-induced alterations in the bone marrow microenvironment. We observed significant statistical differences in these values between the healthy population and lung cancer patients, suggesting their potential as diagnostic markers (Fig. 5A, B, C, D). The nuanced differences between genders further imply that hormonal and genetic factors might modulate these inflammatory markers differently in males and females.

Interestingly, the biomarkers LYMPH, MONO, and NEUT did not show significant differences between lung adenocarcinoma (LUAD), interstitial lung disease (ILD), and complex pulmonary fibrosis (PF), indicating a lack of specificity in these markers for different lung pathologies. This could reflect a common inflammatory pathway activated in various lung diseases, but with distinct molecular triggers and pathways specific to each condition [50]. In contrast, the statistical significance of the PLT indicator between healthy individuals and ILD patients, as well as between LUAD and ILD patients, underscores the potential role of platelets in fibrotic and neoplastic lung diseases. The involvement of platelets in tumour progression is well-documented, with evidence showing that platelets can contribute to tumour growth and metastasis. This occurs through mechanisms such as facilitating tumour cell evasion from the immune system and promoting angiogenesis, both of which are crucial processes in cancer development [29]. A notable statistical difference observed between males and females in ILD patients suggests the influence of sex-specific factors on platelet behaviour and disease pathology. When examining biomarker ratios, namely MLR, NLR, and PLR, we found high statistical significance between healthy individuals and lung cancer patients, emphasizing the importance of these ratios in disease detection (Fig. 5E, F, G). These ratios reflect the balance between pro-tumorigenic inflammation and anti-tumorigenic immune surveillance, offering insights into the tumour microenvironment’s dynamics. Nikolic´ et al. [51] reported that lung cancer patients and healthy individuals significantly differ in their NLR and PLR, but these markers do not distinguish between lung cancer subtypes. This finding suggests that while NLR and PLR are indicative of an overarching cancer-associated inflammatory response, they lack the specificity to differentiate between the molecular and histological heterogeneity of lung cancer subtypes. Our findings corroborate these observations and highlight the need for identifying additional biomarkers that can provide more detailed insights into the biological mechanisms driving different lung diseases.

In addition, we found that both MLR and NLR exhibited significant differences across LUAD, ILD, and complex PF, even when considering gender differences. In contrast, while the PLR indicator displayed no correlation with gender among the various lung conditions, it demonstrated a certain degree of statistical significance when comparing ILD patients with complex PF patients. It should be noted that while the model demonstrates good performance in distinguishing between healthy individuals and those with PF correspond with Jiang et al. [52], it does not perform as well when attempting to differentiate between various lung diseases (Supplementary Fig. 7). This limitation points to the need for additional biomarkers such as smoking history or combined diagnostic strategies for more precise categorisation among various lung conditions.

Our predictive model, which employs a blend of serum indicators and a machine learning algorithm, was engineered for the early detection and diagnosis assessment of lung diseases. The ROC curve outcomes for these ratios in healthy and complex PF patients (Fig. 6) reveal that AGR, MLR, NLR, and PLR ratios all demonstrate admirable predictive capabilities, with corresponding AUC values of 0.642, 0.796, 0.833, and 0.699. Notably, NLR proves to be the most precise indicator in the participants with complex PF. Additionally, among healthy individuals and LUAD patients (Supplementary Fig. 6A), MLR and NLR deliver the most potent predictive power, evidenced by the AUC of 0.775 and 0.758, respectively. In the case of healthy individuals and ILD patients (Supplementary Fig. 6B), MLR and NLR again exhibit the best predictive capability with AUC values of 0.602 and 0.609, respectively. Hence, we can conclude that regardless of the comparison between healthy individuals and lung patients, NLR remains a strong indicator, showcasing robust predictive power.

## Conclusion

In summation, lung diseases are more prevalent among men, whereas female patients display a notable propensity for fibrosis complications and a younger age profile, especially in the ILD-PF patients. Our thorough research has validated serum biomarkers AGR, MLR, NLR, and PLR as pivotal in lung cancer diagnosis. Specifically, NLR stands out for its precision and consistency across various health conditions. This study highlights the important auxiliary role of serum biomarkers and their ratios in predicting lung disease, providing new approaches for early detection and tailored treatment of patients.

## Supplementary Information

Supplementary Material 1.

## Data Availability

The original contributions presented in this study are included in the article/supplementary material, further inquiries can be directed to the corresponding author.
