# Supplementary material for: Novel prediction model of early screening lung adenocarcinoma with pulmonary fibrosis based on haematological index
Source: BMC Cancer. 2024 Sep 27;24:1178. doi: 10.1186/s12885-024-12902-6 (PMC11438419; doi:10.1186/s12885-024-12902-6)
Supplement: Supplementary file 1 — Supplementary Material 1. [file 12885_2024_12902_MOESM1_ESM.docx]

# Contents

**Appendix A. Supplementary Information**

### Novel Prediction Model of Early Screening Lung Adenocarcinoma with Pulmonary Fibrosis based on Haematological Index

Haiyang Li^†^, Xing Fu^†^, Mingtao Liu^†^, Wenhan Cao, Zhiman Liang, Zhangkai J. Cheng^∗^, Baoqing Sun^∗^

Here is the supplementary information for this article. The contents in the supplementary figure are all supplements to the viewpoints and information in the paper. Some of the multi-indicator maps cannot be directly put into the main figure of the text, so they are presented in the supplementary materials, and the expanded contents of this study are discussed more comprehensively.

The Naive Bayes algorithm is a classification technique based on Bayes’ Theorem. We use Bayesian methods to construct disease prediction models in our research. The formula for Bayes’ Theorem is as follows:

*P*(*A* | *B*) = *P*(*B* | *A*)*P*(*A*)

*P*(*B*)

(1)

In the context of Naive Bayes, ’A’ and ’B’ are events and *P*(*B*) ̸= 0. *P*(*A*|*B*) is the posterior probability of class (target) given predictor (attribute). P(A) is the prior probability of class. *P*(*B*|*A*) is the likelihood which is the probability of the predictor given class. P(B) is the prior probability of the predictor.

In statistics, Pearson’s correlation coefficient, symbolized as ’r’, serves as a robust metric assessing both the magnitude and direction of the linear association between two variables. We analysed the correlation of indicators within different diseases through Pearson’s correlation, and the results are shown in Supplementary Figure [1](#_bookmark59). The computation of ’r’ necessitates the insertion of covariance and variance estimates into a designated formula, as detailed subsequently.

*n*

∑

*i*=1

(*x_i_* − *x*¯) (*y_i_* − *y*¯)

*r_xy_* = q *n*

∑*i*=1 (*x_i_* − *x*¯)

2q *n*

(2)

2

where *n* is sample size, *x_i_*,*y_i_* are the individual sample points indexed with *i*, *x*¯ = ^1^ ∑*^n^*

∑*i*=1 (*y_i_* − *y*¯)

*x_i_* (the sample mean); and

analogously for *y*¯.

*n i*=1

The normalisation of data using the Z-score method is commonly carried out to ensure that each feature contributes equally to the analysis. The formula for Z-score normalisation, also known as standardisation, is given by:

*z* = (*x* − *µ*)

*σ*

(3)

where: x is the original data point, *µ* is the mean of the data points, *σ* is the standard deviation of the data points.

For each data point, the Z-score represents how many standard deviations the point is from the mean. This process converts the data to a standard scale without distorting the ranges of values. After normalisation, the mean of the transformed data is 0 and the standard deviation is 1. This is particularly useful in machine learning algorithms that are sensitive to the scale of the data, ensuring that each feature contributes proportionately to the final analysis.

Supplementary Figure [1](#_bookmark59) comprehensively shows the correlation analysis results between blood indicators in the healthy population, LUAD patients, ILD patients and complex PF patients. Supplementary Figure [2](#_bookmark60) shows the correlation distribution among blood indicators in the healthy population, LUAD, ILD and complex PF patients. Supplementary Figure [3](#_bookmark61) shows the complex PF group was divided into different complications, the distribution of patients and the statistical difference analysis of indicators. Supplementary Figure [4](#_bookmark62) shows the distribution of the NUETP/LYHPHP ratio and data fitting results in the complex PF subgroup. Supplementary Figure [5](#_bookmark63) shows the prediction performance of ratio-related indicators in prediction models for 2124 complex PF patients. Supplementary Figure [6](#_bookmark64) shows the prediction performance of indicator ratio in prediction models.

Supplementary Figure [7](#_bookmark65) shows the ROC curves demonstrating the classification performance of the Naive Bayesian model for KL-6 across different disease cohorts. Panel A shows that the AUC for distinguishing between healthy individuals and patients with pulmonary fibrosis (PF) using KL-6 is 0.781. This indicates a good level of discriminative ability, suggesting that KL-6 is a potential biomarker for identifying PF.In Panel B, the AUC for differentiating between interstitial lung disease (ILD) and PF is 0.598. This lower AUC suggests that while KL-6 may be indicative of lung pathology, its levels do not as clearly differentiate between ILD and PF. Panel C presents an AUC of 0.587 for distinguishing between lung adenocarcinoma (LUAD) and PF. Similar to ILD vs PF, the AUC indicates moderate discrimination, implying that KL-6 levels alone may not be sufficient to distinguish between LUAD and PF.

The trend in KL-6 levels appears to be more significantly associated with PF when compared to healthy controls, as evidenced by the higher AUC in Panel A. However, when it comes to differentiating PF from other lung pathologies such as ILD and LUAD, the discriminative power of KL-6 decreases, as shown by the lower AUC values in Panels B and C. This suggests that while KL-6 is a promising biomarker for PF, its specificity may be limited when distinguishing between different lung diseases, particularly those with similar pathological features or those that may co-occur with PF. The model’s performance and the AUC values indicate that additional biomarkers or clinical parameters might be necessary to improve the specificity and sensitivity of disease classification in a clinical setting.

Supplementary Figure [8](#_bookmark66) shows the ROC (Receiver Operating Characteristic) curves for the Naive Bayes classifier at two different training set sizes, 70% and 80%, illustrating the model’s ability to distinguish between classes under varied conditions. Supplementary Figure [8](#_bookmark66)A illustrates that the Monocyte-to-Lymphocyte Ratio (MLR) model yields an AUC of 0.775 on a 70% training set. This suggests that the model has good predictive accuracy when a larger portion of the data is used for training. Supplementary Figure [8](#_bookmark66)B shows a slight improvement in the AUC to 0.778 when the training set is increased to 80%, indicating that a larger training set marginally enhances the model’s ability to generalise and predict the test data. Supplementary Figure [8](#_bookmark66)C presents a comparison of different models. The Naive Bayesian (NB) model has an AUC of 0.777, the Random Forest (RF) model shows a slightly better AUC of 0.792, the K-Nearest Neighbours (KNN) model significantly lags behind with an AUC of 0.555, and the Support Vector Machine (SVM) has an AUC of 0.720. The superior performance of the NB model (especially compared to KNN and SVM) suggests that it effectively captures the underlying distributions of the data even with the assumption of feature independence. Its success over other models may also be due to its simplicity and the specific way it handles categorical data, which can sometimes be advantageous depending on the nature of the dataset. Despite RF having the highest AUC, the NB model’s performance is quite comparable, and it might be preferred for its simplicity, interpretability, and less computationally intensive nature.

The choice of the NB model as the most optimal could be attributed to several factors: it performs well with small datasets, it’s less prone to overfitting compared to more complex models like RF, and it requires a smaller amount of training data to make predictions which is advantageous when data is scarce. Furthermore, the NB model is robust to noise and irrelevant features, which might be present in the dataset. It is particularly effective for classification tasks where the assumption of feature independence holds true or when the cost of false positives and false negatives is not significantly different.

**Table S1.** Statistical table of basic blood test indicators of participants

| Variable | Healthy N=7,137 | LUAD N=7,762 | ILD N=7,955 | complex PF N=2,124 |
| --- | --- | --- | --- | --- |
| AGE | 40±15 | 63±13 | 58±15 | 56±16 |
| TP | 76 (73, 79) | 66 (62, 71) | 65 (60, 71) | 70 (64, 75) |
| ALB | 44.6 (42.6, 46.6) | 36.7 (33.4, 39.8) | 35.9 (32.5, 39.2) | 39.4 (35.7, 42.5) |
| hs-CRP | 1 (1, 2) | 6 (1, 29) | 5 (1, 17) | 4 (1, 9) |
| CRP | 0.2 (0.1, 0.4) | 1.6 (0.4, 7.2) | 0.4 (0.1, 1.4) | 0.5 (0.1, 1.6) |
| CEA | 2 (1, 3) | 4 (2, 10) | 4 (2, 7) | 3 (2, 8) |
| NSE | 13 (12, 17) | 18 (14, 24) | 18 (15, 24) | 15 (12, 19) |
| NEUTP | 58 (52, 64) | 69 (61, 78) | 77 (64, 87) | 67 (57, 78) |
| LYMPHP | 32 (27, 38) | 19 (12, 26) | 14 (6, 24) | 22 (13, 31) |
| PLT | 245 (208, 288) | 234 (187, 298) | 204 (144, 270) | 215 (164, 268) |
| MONOP | 6.6 (5.4, 7.8) | 8.0 (6.1, 10.0) | 6.7 (4.2, 9.0) | 7.2 (5.3, 9.5) |
| NEUT | 3.9 (3.1, 4.9) | 5.4 (3.8, 7.6) | 6.4 (4.3, 9.5) | 4.7 (3.2, 7.6) |
| LYMPH | 2.2 (1.8, 2.7) | 1.4 (1, 1.9) | 1.2 (0.6, 1.9) | 1.5 (1, 2) |
| MONO | 0.5 (0.4, 0.6) | 0.6 (0.4, 0.8) | 0.6 (0.4, 0.8) | 0.5 (0.4, 0.8) |
| WBC | 6.9 (5.8, 8.2) | 7.9 (6.2, 10.2) | 9.2 (6.5, 12.8) | 7.4 (5.4, 10.4) |
| RDW | 3.0 (12.5, 13.6) | 4 (13.3, 15.3) | 15 (13.8, 17.2) | 14 (13.1, 15.5) |
| K | 4.14 (3.97, 4.35) | 3.89 (3.62, 4.16) | 3.86 (3.56, 4.19) | 3.97 (3.69, 4.24) |
| Ca | 2.39 (2.32, 2.47) | 2.18 (2.1, 2.27) | 2.17 (2.07, 2.26) | 2.24(2.13, 2.34) |
| Na | 139.8 (138.2, 141.2) | 137.8 (135.7, 139.7) | 138.4 (136.0, 140.9) | 138.7(136.4, 140.7) |
| AGR | 1.43 (1.3, 1.6) | 1.25 (1.06, 1.44) | 1.22 (1.01, 1.45) | 1.29(1.14, 1.45) |
| NLR | 2 (1, 2) | 4 (2, 6) | 6 (3, 14) | 3 (2, 6) |
| MLR | 0.21 (0.16, 0.26) | 0.43 (0.28, 0.67) | 0.47 (0.27, 0.88) | 0.33 (0.22, 0.57) |
| PLR | 112 (89, 140) | 165 (117, 244) | 175 (109, 309) | 142 (100, 213) |
| KL-6 | 225 (155, 288) | 457 (290, 850) | 939 (483, 1752) | 770 (441, 1462) |


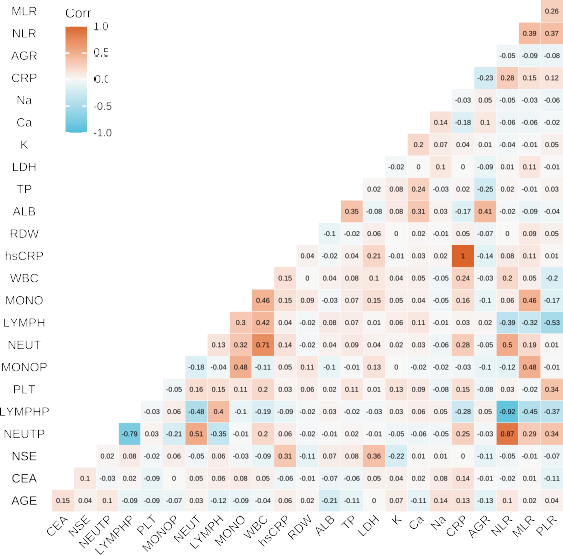
**A** Healthy **B** LUAD


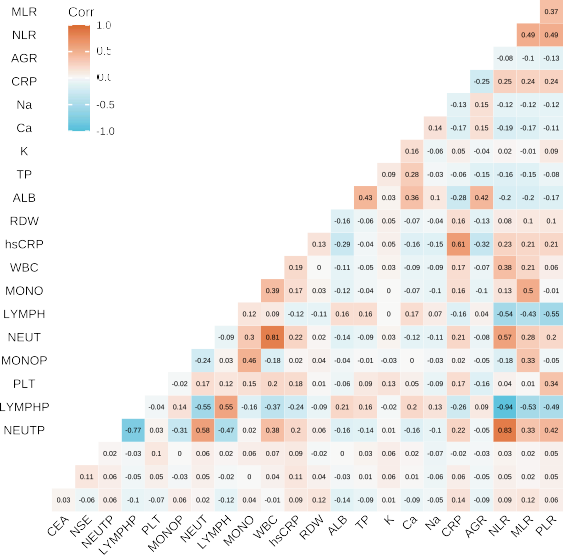

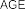

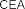

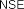


**D**


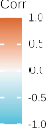

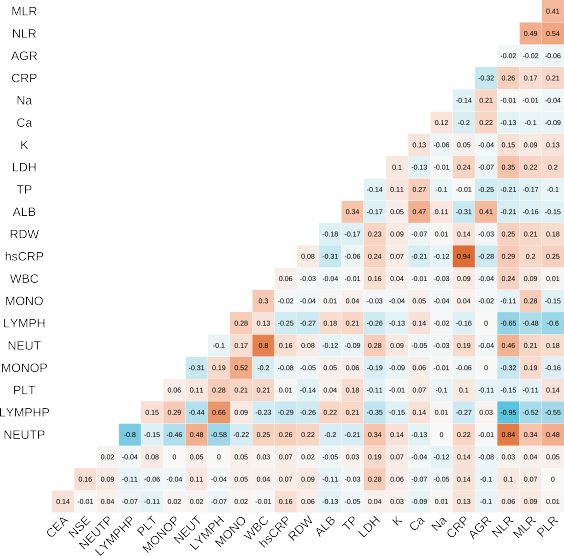

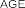

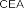

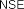


**C**

ILD

PF


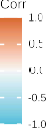

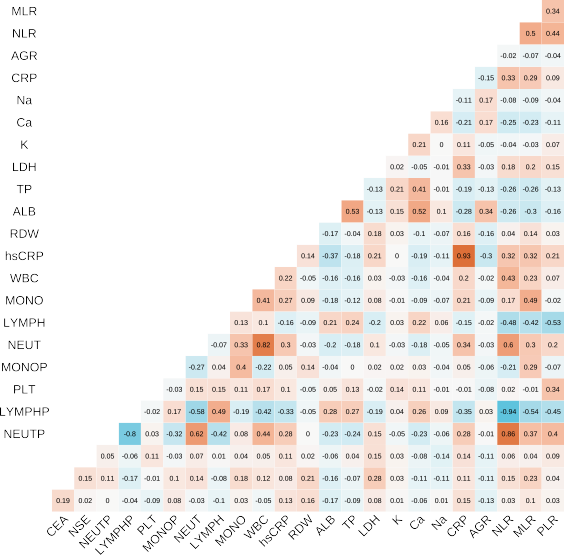

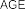

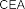

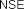


**Supplementary Figure 1.** The correlation analysis results between blood indicators in **A**) the healthy population, **B**) LUAD patients, **C**) ILD patients, **D**) complex PF patients.





**Supplementary Figure 2.** The correlation distribution among blood indicators in the healthy population, LUAD, ILD and complex PF patients. In this paper, we have compared and discussed in detail the scattered distribution relationship of AGR, NLR, PLR and MLR in different types of patients. In the supplementary materials, the relationship between more indicators is visualized in detail, and in addition, we can also understand that the scattered distribution of indicators between different genders can affect the distribution of beans in this paper. At the same time, it is interesting to visualize the distribution between scales.

##
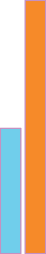

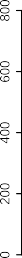
A B C D


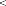

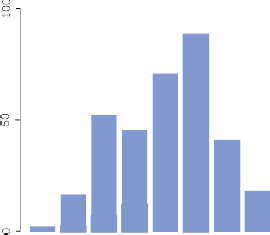

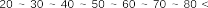


**L-I-PF**


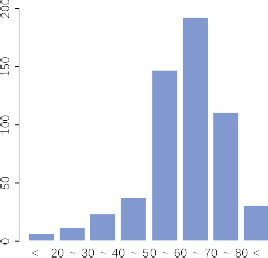


**LUAD-PF**


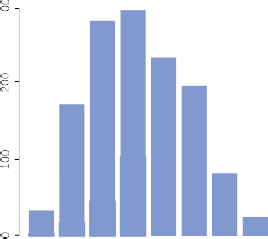

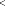

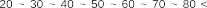


**ILD-PF**

Cases

3


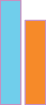

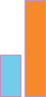


**LUAD-PF**


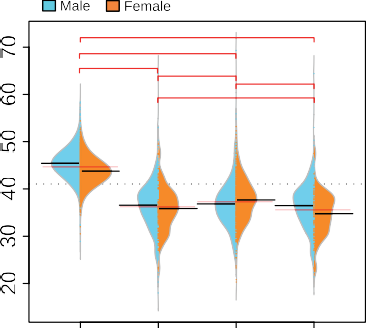

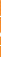

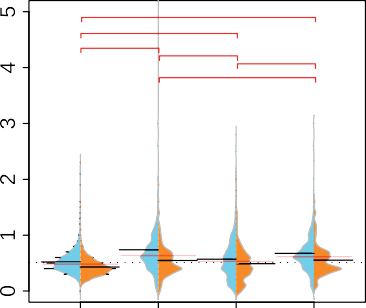

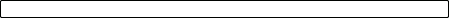


***

*** ***

*

**Statistical significance** Total  ~~*~~  Gender

F

ns **

*

**

**

*

ns *

**Healthy LUAD-PF**

** *

**ILD-PF**

*

**L-I-PF**

**

**Healthy LUAD-PF**

*

**

**ILD-PF**

*

**L-I-PF**

*

## E

ALB (mg/mL)

**ILD-PF**

**L-I-PF**

## G


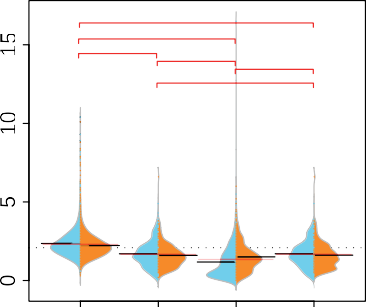


** **

*

*

ns *

ns

ns

*

ns

*

MONO (mol/L)

LYMPH (mol/L)

H I J


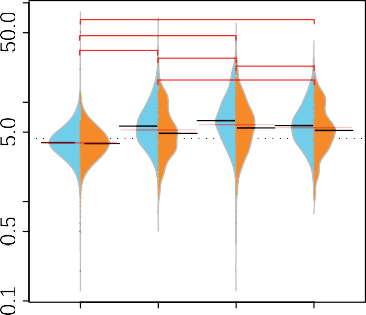

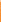


**

*** ***

*

ns

ns

ns

**

**

*


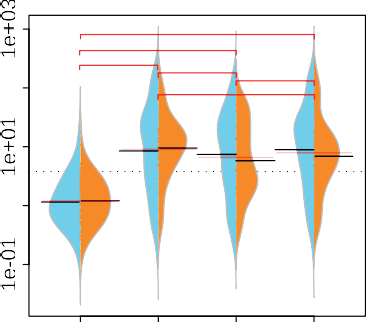


*** *** ***

*

*

*

ns

**Healthy LUAD-PF ILD-PF**

* *

**L-I-PF**

*

hs-CRP (mg/mL)

PLT (mol/L)

**Healthy LUAD-PF ILD-PF L-I-PF**

**Healthy LUAD-PF ILD-PF L-I-PF**


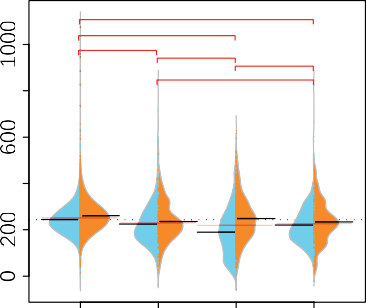


*

**

*

ns

ns

ns

**Healthy LUAD-PF ILD-PF**

*

* ***

**L-I-PF**

*

NEUT (mol/L)

**Supplementary Figure 3.** The complex PF group was divided into different complications (LUAD-PF, ILD-PF, L-I-PF), **B-D**) the distribution of patients **E-J**) the statistical difference analysis of indicators. For 2124 patients with LUAD or ILD complicated with PF, we subdivide the different complications, namely 615 patients with LUAD-PF,

1228 patients with ILD-PF, and 281 patients with LUAD-ILD-PF (L-I-PF). The distribution of patients is shown in the supplementary material Figure 3A, and the age distribution of patients in different groups is shown in B-D. In addition, we compared the index differences between each group and the healthy population. We found that there was no significant difference in related indexes between patients with fibrosis, but there were extremely significant statistical differences between them as shown in E-J.

## A


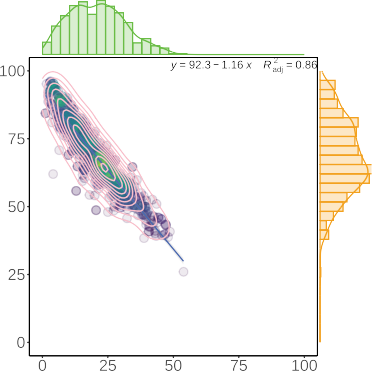


**LUAD-PF**


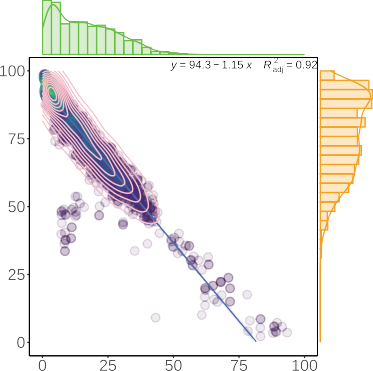


B

**ILD-PF**


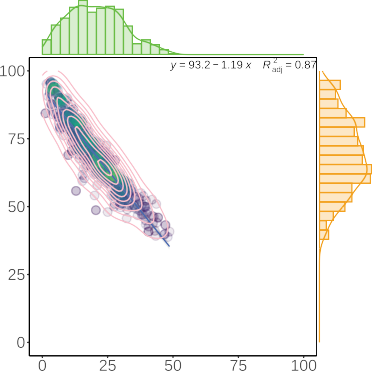


**L-I-PF**

C

LYMPHP (%)

NUETP (%)

NUETP (%)

NUETP (%)

LYMPHP (%)

LYMPHP (%)

**Supplementary Figure 4.** The distribution of NUETP/LYHPHP ratio and data fitting results in the complex PF subgroup. **A**) LUAD-PF, **B**) ILD-PF, **C**) L-I-PF. We discussed NUETP/LYHPHP in detail in the text and found that NLR is the best predictor among various indicators. Therefore, we also discussed the internal consolidation group of complex PF in the supplementary materials. It can be seen that the distribution of groups and LUAD-PF concentration of patients (NUETP: 0.75, LYHPHP: 0.25) and ILD-PF (NUETP: 0.9, LYHPHP: 0.05) are basically consistent with the distribution of LUAD and ILD in the text.


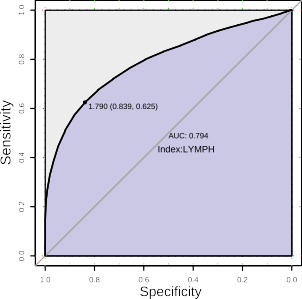

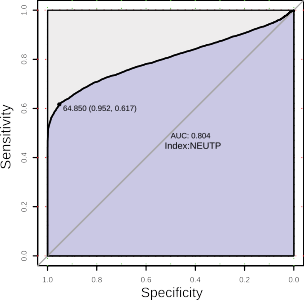

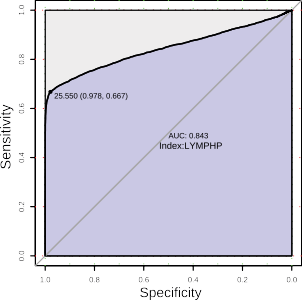


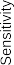

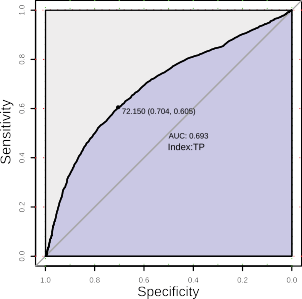

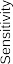

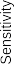

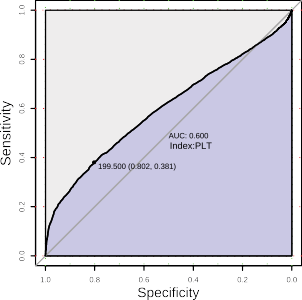


**Supplementary Figure 5.** The prediction performance of ratio-related indicators in prediction models for 2124 complex PF patients. While conducting model prediction performance tests on the proportion, we also conducted corresponding tests on other indicators. The Receiver operating characteristic of each indicator is shown in the figure. Indicators such as NEUTP and LYMPHP already have good ROC prediction performance before combining ratios, so they have better specificity after proportion merging.


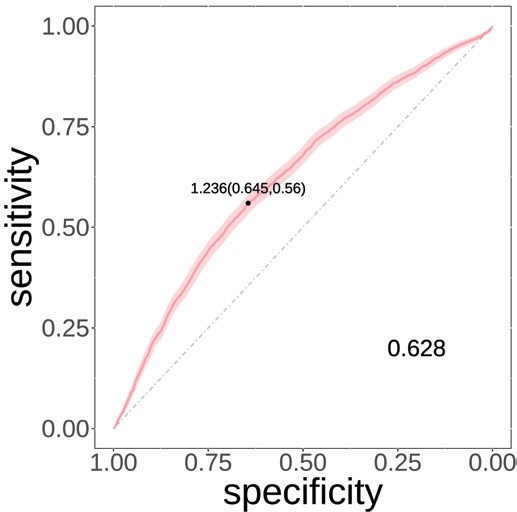

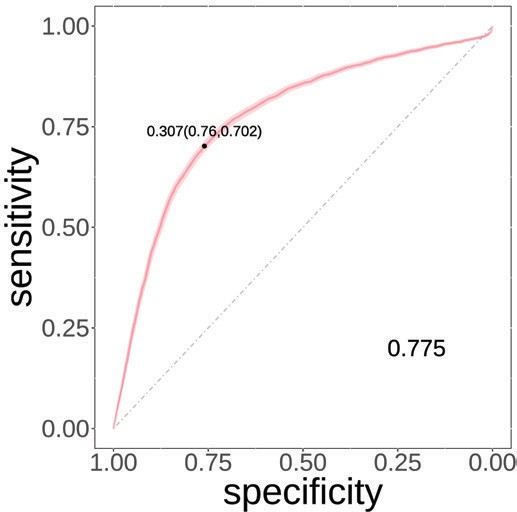

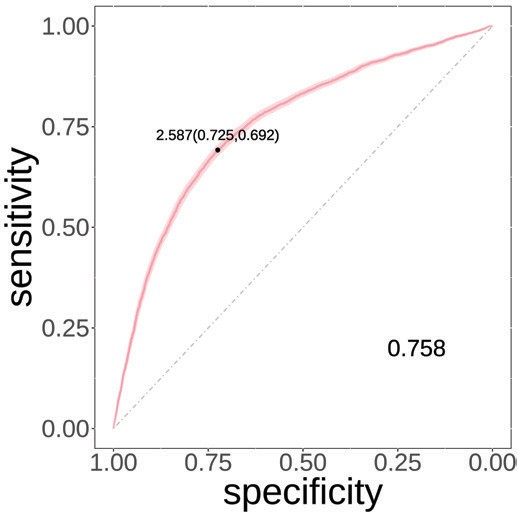

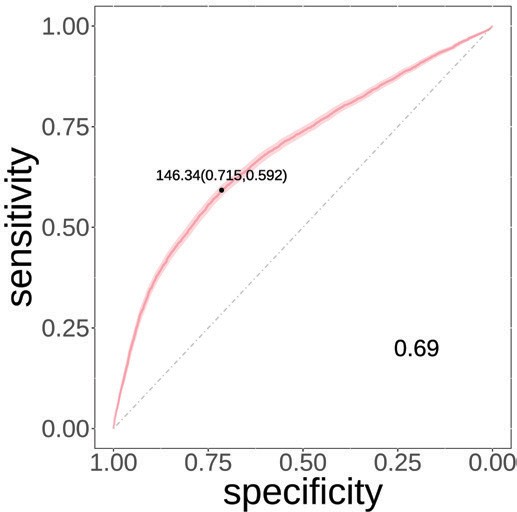


A

AGR

AUC:

MLR

AUC:

NLR

AUC:

PLR

AUC:


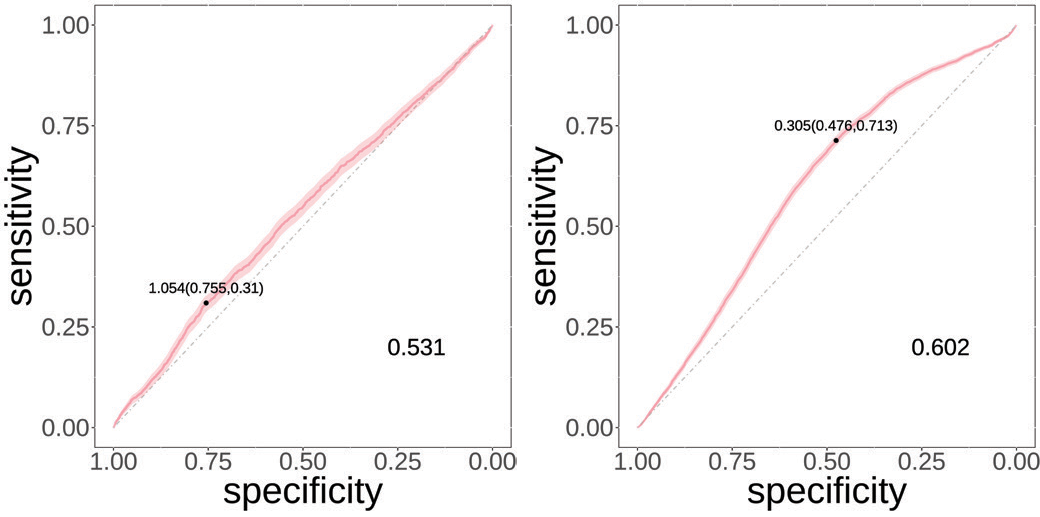

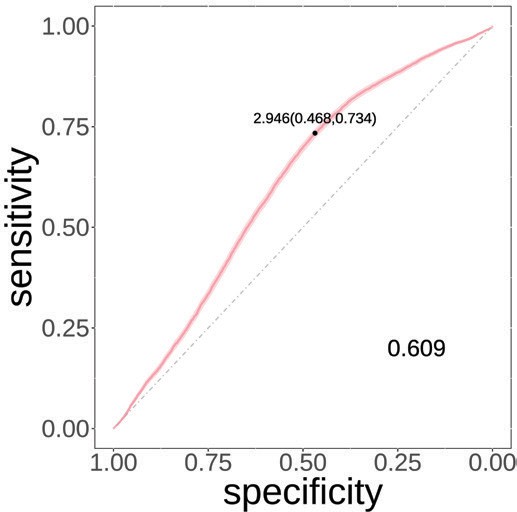

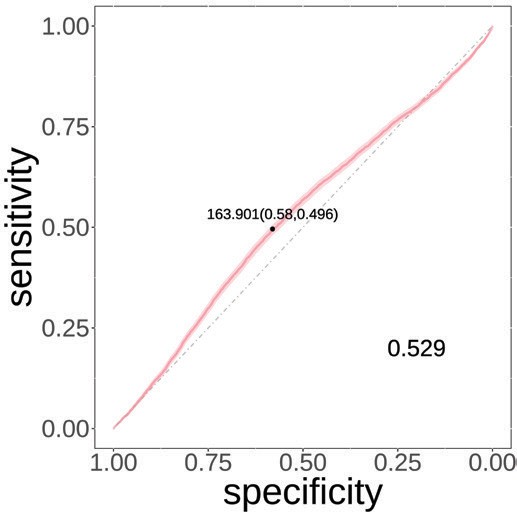


B

AGR

AUC:

MLR

AUC:

NLR

AUC:

PLR

AUC:

**Supplementary Figure 6.** The prediction performance of indicator ratio in prediction models. **A**) Healthy and LUAD patients. **B**) Healthy and ILD patients. Our model also tested the indicators of the predictive performance of diseases such as LUAD and ILD. Among them, the proportion of indicators has a good prediction performance for LUAD. MLR and NLR have good performance. Relative models are not ideal for the performance of ILD. The performance of ILD is not ideal. The best NLR index performance of all proportions is only 0.609.


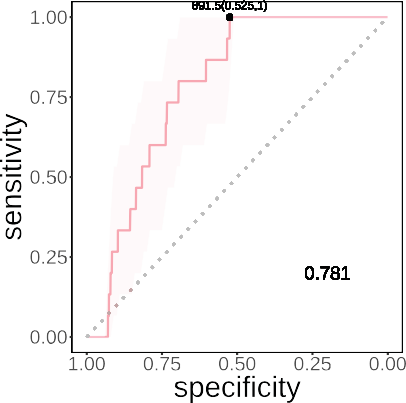


A

KL-6

AUC:

Health vs PF


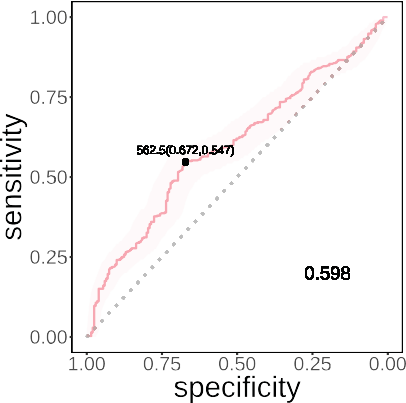


B

KL-6

AUC:

ILD vs PF


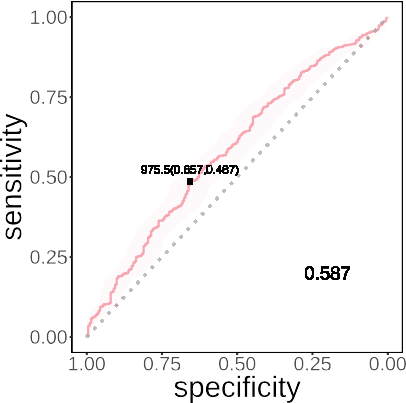


C

KL-6

AUC:

LUAD vs PF

**Supplementary Figure 7.** The ROC curves demonstrating the classification performance of the Naive Bayesian model for KL-6 across different disease cohorts **A**) Health vs. PF: KL-6 Discrimination, **B**) ILD vs. PF: KL-6 Discrimination, and **C**) LUAD vs. PF: KL-6 Differentiation


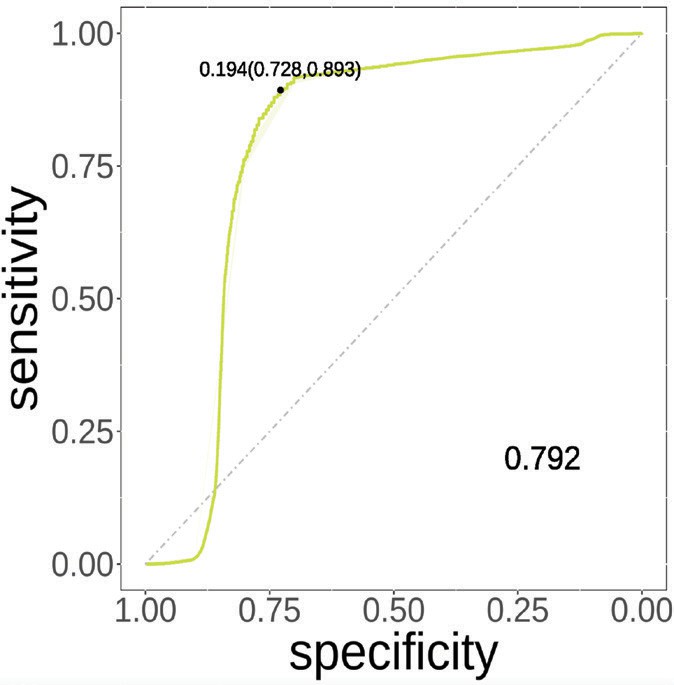

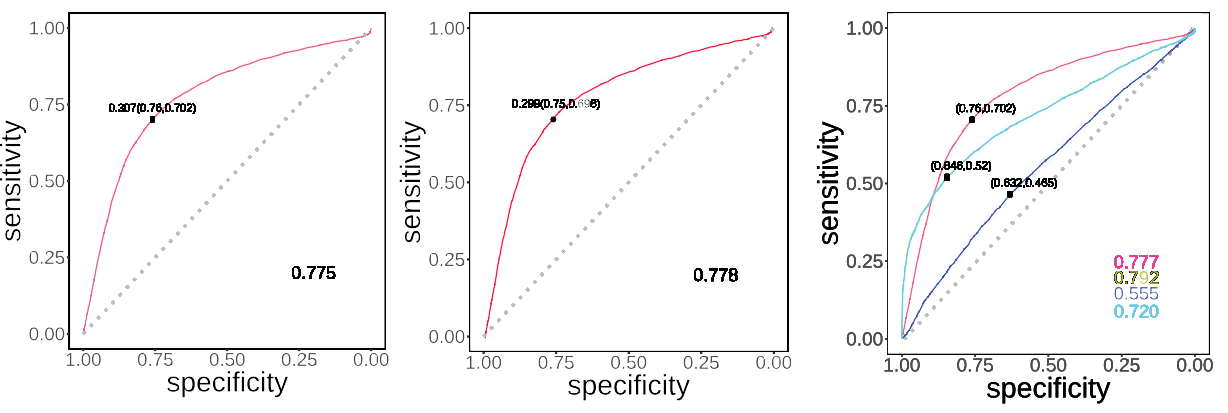


A

70% Train set

B

80% Train set

C

(0.728,0.893)

MLR

AUC:

MLR

AUC:

AUC

NB:

RF: KNN: SVM:

**Supplementary Figure 8.** Comparative analysis of classification model performances with varying training set sizes and among different classifiers. **A**) Performance of naive Bayes classifier with 70% training set. **B**) Performance of naive Bayes classifier with 70% training set. **c**) Comparative performance of multiple classification models, where NB is Naive Bayesian classification, RF is Random forest, KNN is K-Nearest neighbours algorithm, and SVM is support vector machine algorithm.
